# Dual-pathway targeted therapy for decompensated cirrhosis: Combined transjugular intrahepatic portosystemic shunt and cyclooxygenase-2 inhibition

**DOI:** 10.1515/jtim-2026-0077

**Published:** 2026-08-24

**Authors:** Yanting Ye, Zhiyin Huang, Jinhang Gao, Chengwei Tang

**Affiliations:** Department of Gastroenterology, West China Hospital, Sichuan University, Chengdu, Sichuan Province, China

## Introduction

Decompensated cirrhosis is a life-threatening condition primarily manifested by portal hypertension (PHT) and its complications. Transjugular intrahepatic portosystemic shunt (TIPS) has emerged as a crucial therapeutic strategy for PHT complications. However, clinical observations indicate that liver function may continue to deteriorate despite adequate portal pressure control.

The progression of cirrhosis involves two interconnected pathophysiological mechanisms: PHT-driven vascular remodeling and immune dysfunction-mediated chronic systemic inflammation. These parallel mechanisms mutually reinforce each other.^[1]^ Although TIPS effectively control PHT, it does not address the inflammatory component. Cyclooxygenase-2 (COX-2), a key inflammatory mediator, represents a potential therapeutic target. The basic researches have demonstrated that COX-2 inhibition improves experimental liver fibrosis through multiple mechanisms.^[2, 3, 4, 5, 6, 7, 8]^ Furthermore, clinical investigation has shown that combining COX-2 inhibitors with TIPS not only reduces decompensation events but also improves liver function.^[9]^

Building on these observations, we propose a dual-pathway therapeutic strategy combining TIPS with COX-2 inhibitors for decompensated cirrhosis. This article outlines the theoretical foundation, mechanistic evidence, and clinical validation of this novel approach.

## Achievements and limitations of TIPS

TIPS creates an intrahepatic shunt that effectively reduces portal pressure. This procedure demonstrates substantial efficacy in managing variceal bleeding and refractory ascites, leading to reduced rebleeding rates, improved ascites control, and enhanced nutritional status and quality of life. The benefits of TIPS extend to patients with hepatic hydrothorax, hepatocellular carcinoma, and hepatorenal syndrome. Additionally, TIPS serves as an effective bridge therapy before liver transplantation. The implementation of early TIPS strategy has further expanded its therapeutic potential, offering better control of bleeding and ascites while reducing postoperative complications and improving survival.^[10]^

Despite these achievements, TIPS has notable limitations. While effective in controlling PHT-related complications, its impact on overall survival remains controversial, with survival benefits observed only in select patient populations. More importantly, TIPS demonstrates limited ability to improve liver function. Some studies have documented progressive elevation in Model for End-stage Liver Disease (MELD) scores post-TIPS,^[11]^ indicating that portal pressure reduction alone may not be sufficient to enhance hepatocyte function. These observations suggest that effective management of decompensated cirrhosis requires interventions beyond portal pressure reduction, specifically targeting the underlying pathophysiological processes of liver dysfunction.

## Dual pathophysiological mechanisms of liver cirrhosis

Two interconnected mechanisms drive cirrhosis progression: PHT-induced vascular remodeling and immune dysfunction-associated systemic inflammation (Figure 1).

**Figure 1:**
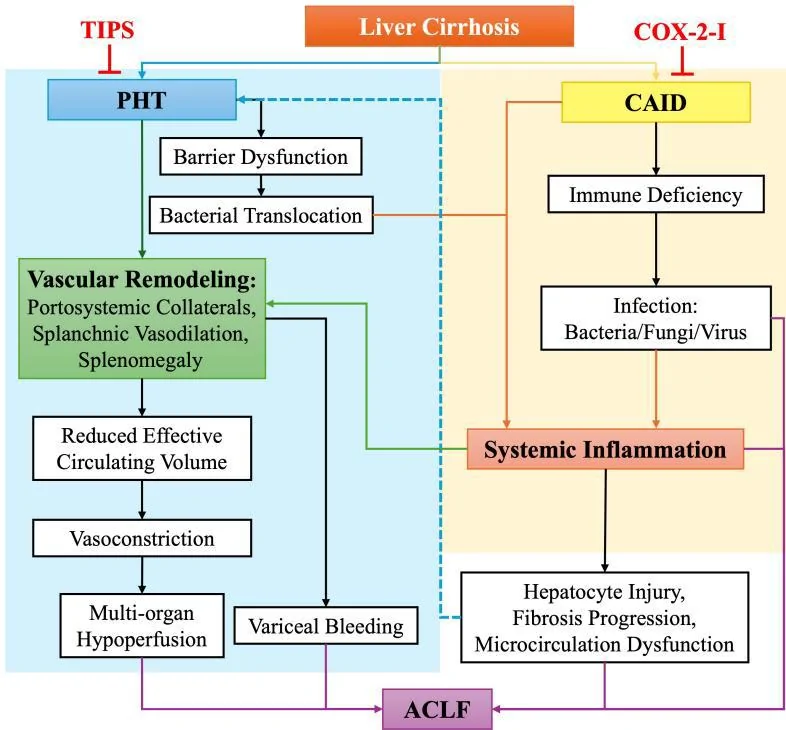
Dual pathophysiological pathways of liver cirrhosis. In the portal hypertension (PHT) pathway (left panel, blue background), portal hypertension leads to barrier dysfunction and subsequent bacterial translocation, which contributes to systemic inflammation. Concurrently, PHT induces vascular remodeling characterized by portosystemic collateral formation, splanchnic vasodilation, and splenomegaly. This vascular remodeling results in reduced effective circulating volume, triggering vasoconstriction, ultimately leading to multi-organ hypoperfusion. Additionally, vascular remodeling can cause variceal bleeding. Transjugular intrahepatic portosystemic shunt (TIPS) directly targets this pathway by reducing portal pressure. In the cirrhosis-associated immune dysfunction (CAID) pathway (right panel, orange background), immune deficiency predisposes patients to infections, which further promote systemic inflammation. The systemic inflammation acts as a central mediator, exacerbating vascular remodeling and driving hepatocyte injury, fibrosis progression, and microcirculation dysfunction. Cyclooxygenase-2 inhibitor (COX-2-I) targets this pathway by modulating immune dysfunction and systemic inflammation. These pathophysiological processes finally culminate in acute-on-chronic liver failure (ACLF) (bottom, purple box). Dotted lines indicate feedback loops where hepatocyte injury, fibrosis progression, and microcirculation dysfunction can further aggravate portal hypertension, perpetuating the pathological cycle. The combination of TIPS and COX-2-I provides dual-pathway intervention, addressing both mechanical and inflammatory mechanisms of disease progression.

In cirrhosis, increased intrahepatic vascular resistance from fibrosis, stellate cell activation, and endothelial dysfunction initiates PHT. Subsequent upregulation of angiogenic factors drives vascular remodeling, manifesting as portosystemic collaterals, splanchnic vasodilation, and splenomegaly. Beyond causing varices and organ hypoperfusion, elevated portal pressure compromises intestinal barrier function, leading to bacterial translocation and systemic inflammation. This inflammatory response further enhances vascular remodeling, establishing a self-perpetuating cycle that often progresses to severe complications like hepatorenal syndrome and acute-on-chronic liver failure.

Cirrhosis-associated immune dysfunction syndrome (CAID) represents another fundamental pathological process, characterized by concurrent systemic inflammation and immune deficiency. Bacterial products and cellular debris from liver injury trigger inflammatory cytokine production. The resulting chronic inflammation damages liver cells, promotes vascular remodeling, and accelerates fibrosis.

The concurrent immune deficiency increases infection susceptibility, which further intensifies inflammation, creating a cycle of immune impairment, infection, inflammation, and organ injury.^[1]^

These two pathophysiological pathways develop in parallel and interact with each other. While TIPS effectively reduces portal pressure, it cannot address systemic inflammation from immune dysfunction. Furthermore, TIPS-mediated portal blood shunting bypasses hepatic immune filtering, potentially aggravating inflammation. This explains why some patients show continued liver deterioration and increased infection risk despite portal pressure control, limiting survival benefits.

## COX-2 inhibitors: a targeted weapon against inflammation

COX-2 plays a key role in hepatic inflammation and fibrosis progression, with its expression significantly increasing during chronic liver injury and correlating with fibrosis severity. The therapeutic mechanisms of COX-2 inhibitors in cirrhosis have been systematically investigated.

Celecoxib, a selective COX-2 inhibitor, improves PHT by inhibiting VEGF/VEGFR-2 expression to reduce pathological angiogenesis, suppressing hepatic stellate cell activation to alleviate fibrosis, and increasing nitric oxide bioavailability to improve sinusoidal endothelial cell function.^[2]^ At the cellular level, celecoxib provides multiple protective effects. It reduces hepatocyte apoptosis by inhibiting endoplasmic reticulum stress,^[3]^ decreases ferroptosis by upregulating antioxidant systems,^[2,4]^ and maintains normal hepatocyte function by blocking epithelial-mesenchymal transition.^[5]^ Recent finding showed that COX-2 inhibition can correct abnormal hepatocyte-cholangiocyte transdifferentiation through the TGF-β-TGFBR1-β-catenin pathway, improving liver function.^[6]^ Importantly, celecoxib targets inflammation through gut-liver^[7]^ and spleen-liver axes.^[8]^ It maintains intestinal barrier integrity to prevent bacterial translocation while suppressing splenic inflammatory responses to reduce pro-inflammatory mediators in cirrhosis.

## Clinical evidence for dual-pathway targeted therapy

Based on the complementary effects of TIPS-mediated hemodynamic control and COX-2 inhibitor-induced protective actions, a dual-pathway targeted therapy for decompensated cirrhosis is emerging. To validate this innovative approach, we conducted a single-center retrospective cohort study of cirrhotic patients with concomitant rheumatoid arthritis or osteoarthritis who underwent TIPS. The study compared 51 post-TIPS patients receiving long-term COX-2 inhibitors at half of the standard dose (≥ 6 months) with 89 TIPS-only controls. Primary endpoint was hospitalization-requiring decompensation events, with secondary endpoints including modified albumin-bilirubin (mALBI) score and renal function. The cumulative incidence of decompensation events was significantly lower in the TIPS plus COX-2 inhibitor group compared to TIPS alone, showing nearly a threefold reduction. Multivariate analysis identified COX-2 inhibitor use as an independent protective factor. Furthermore, the combination group exhibited lower shunt dysfunction rates, possibly because the anti-inflammatory effects of COX-2 inhibitor on the shunt endothelium mitigated neointimal hyperplasia. This group was also the only one to achieve a significant improvement in the mALBI score, particularly in grade 2b patients, suggesting early intervention benefits for liver function.

Safety is a critical consideration. No major adverse events, such as gastrointestinal bleeding, cardiovascular events, or renal impairment, were observed in the cirrhotic patients with long-term administration of a low dose of COX-2 inhibitor. Though nonsteroidal anti-inflammatory drugs (NSAIDs) may impair renal perfusion in patients with decompensated cirrhosis, TIPS placement likely mitigates this risk by improving effective circulating volume and reducing renin-angiotensin-aldosterone system (RAAS) and sympathetic nervous system (SNS) activity, making the kidney less susceptible to COX-2 inhibition. Nonetheless, this approach warrants caution, and diligent renal function monitoring is essential.

There are several limitations of this study. The relatively small sample size, potential selection bias, and lack of etiology-based stratification may limit the generalizability of those findings, underscoring the necessity for prospective validation in a broader cirrhotic population.^[9]^

## Conclusions and future perspectives

The cumulative studies demonstrate that combining TIPS with COX-2 inhibitors simultaneously improves PHT and systemic inflammation. The pioneering translational medical investigation suggests that effective management of decompensated cirrhosis requires both TIPS for acute complications and vascular remodeling, and COX-2 inhibitors for inflammation control and liver protection. This mechanism-based comprehensive intervention approach represents a shift from passive complication management to active pathophysiological intervention in cirrhosis treatment.

The value of this innovative strategy extends beyond post-TIPS management. This dual-pathway treatment offers new possibilities for preventing disease progression, reducing complications, and improving long-term outcomes. The observed liver function improvement challenges the concept of irreversible decompensation, suggesting that combined mechanical and pharmacological interventions might delay or partially reverse disease progression. This concept could be applied beyond TIPS to other PHT treatments, including endoscopic and pharmacological therapies, potentially initiating a new era in comprehensive cirrhosis management.

Despite encouraging evidence, several challenges remain. Large-scale prospective studies are essential to validate the safety and efficacy of this strategy. Future studies must define optimal patient selection criteria, likely excluding those with significant renal impairment. Optimal intervention timing and individualized treatment protocols also require careful investigation. Understanding molecular response mechanisms may help identify predictive biomarkers for personalized therapy.

The role of this strategy in special populations, such as patients with different etiologies or those awaiting liver transplantation, deserves particular attention. Additionally, investigating its impact on portal vein thrombosis, hepatocellular carcinoma prevention, and quality of life could reveal broader therapeutic benefits.

As our understanding of cirrhosis pathophysiology evolves, the combination with TIPS and COX-2 inhibitor represents a promising step toward mechanism-based therapy. This approach may ultimately transform cirrhosis management from passive complication control to active disease modification, potentially improving the natural history of this challenging condition.
